# Reforestation regulated soil bacterial community structure along vertical profiles in the Loess Plateau

**DOI:** 10.3389/fmicb.2023.1324052

**Published:** 2023-11-28

**Authors:** Fan Wu, Yunqiang Wang, Hui Sun, Jingxiong Zhou, Ruijie Li

**Affiliations:** ^1^Key Laboratory of Ecosystem Network Observation and Modeling, Institute of Geographic Sciences and Natural Resources Research, Chinese Academy of Sciences, Beijing, China; ^2^University of Chinese Academy of Sciences, Beijing, China; ^3^State Key Laboratory of Loess and Quaternary Geology, Institute of Earth Environment, Chinese Academy of Sciences, Xi'an, Shaanxi, China; ^4^Xi’an Institute for Innovative Earth Environment Research, Xi’an, China

**Keywords:** ecological reforestation, stand age, deep soil, bacterial community structure, bacterial function

## Abstract

**Introduction:**

Reforestation is a widely used strategy for ecological restoration in areas facing ecological degradation. Soil bacteria regulate many functional processes in terrestrial ecosystems; however, how they respond to reforestation processes in surface and deep soils remains unclear.

**Methods:**

Artificial *Robinia pseudoacacia* plantation with different stand ages (8, 22, and 32 years) in a typical fallow forest on the Loess Plateau was selected to explore the differential response of soil bacterial community to reforestation in different soil depths (surface 0–200 cm, middle 200–500 cm, and deep 500-100 cm). Soil bacterial diversity, community composition and the co-occurrence patterns, as well as the functions were analyzed.

**Results and discussion:**

The results showed that alpha diversity and the presence of biomarkers (keynote species) decreased with the increasing soil depth, with a sharp reduction in family-level biomarker numbers in 500–1,000 cm depth, while reforestation had a positive impact on bacterial alpha diversity and biomarkers. Reforestation induced a more loosely connected bacterial community, as evidenced by an increase of 9.38, 22.87, and 37.26% in the average path length of the co-occurrence network in all three soil layers, compared to farmland. In addition, reforestation reduced the hierarchy and complexity but increased the modularity of the co-occurrence network in top and deep soil layers. Reforestation also led to enrichment in the relative abundance of functional pathways in all soil layers. This study sheds light on the strategies employed by deep soil bacteria in response to reforestation and underscores the significant potential of deep soil bacteria in terrestrial ecosystems, particularly in the context of human-induced environmental changes.

## 1 Introduction

Reforestation is the predominant ecological restoration strategy to improve ecosystem services (Chazdon, 2008; Hua et al., 2016; Nunes et al., 2020). Specifically, artificial forestation is one of the important ecological managements on the Loess Plateau (Jin et al., 2011). The large-scale reforestation in the past 20 years has effectively mitigated soil erosion and greatly improved the natural ecological environment of the region (Liang et al., 2022). These efforts have brought about substantial benefits to soil structure, soil fertility, carbon sequestration, water balance, and biodiversity (Chazdon, 2008; Cunningham et al., 2015). The age of a forest, i.e., stand age, is a key determinant in reforestation, and it can influence the soil nutrient distribution by altering forest material fractions (Lucas-Borja et al., 2016; Jonsson et al., 2020). Previous studies have shown that the stocks of soil organic carbon and total nitrogen in the forest soil decrease or continue to increase after reaching the peak throughout the reforestation process (Guo and Gifford, 2002; Xu et al., 2019).

As the most crucial and sensitive biological component of the soil ecosystem, soil bacteria regulate most biogeochemical reactions and play a dominant role in the nutrient-cycling process in terrestrial ecosystems (Falkowski et al., 2008; Nelson et al., 2016). Notably, soil bacteria are the main driving factors of organic carbon turnover. Vegetation restoration leads to changes in bacterial community structure by altering plant community composition and soil physicochemical properties (Probst et al., 2018), which in turn affects functional processes such as organic carbon turnover.

A large population of bacteria are concentrated in deep soils, and these deep-lying bacteria play an extremely important role in soil formation, ecosystem geochemical cycling, pollutant degradation, and maintenance of groundwater quality (Falkowski et al., 2008; Hartmann et al., 2009; Eilers et al., 2012; Li et al., 2014; Probst et al., 2018). Nevertheless, most current studies have primarily focused on the surface layer (0–20 cm), where bacterial activity and diversity are the highest (Fierer et al., 2003). Soil bacterial diversity and composition vary with soil depth (Li et al., 2014), and changes in resource and environmental gradients in the soil profile lead to a shift in bacterial communities with soil depth (Chu et al., 2016). The responses of surface and non-surface soil bacteria to reforestation were dissimilar. Previous studies have investigated how reforestation impacts surface soil bacteria (Zheng et al., 2005; Jourgholami et al., 2019; Shao et al., 2019) by altering surface soil bacterial community and biomass (Shao et al., 2019), community composition (Jiao et al., 2018), diversity (Chen et al., 2020), and bacterial functional stability (Jiao et al., 2022). However, much less attention has been paid to the variations of deep soil bacterial community characteristics during reforestation. The response of deep soil bacterial diversity/community composition to the ecological reforestation process remains less clear.

The objective of this study was to investigate the responses of deep soil bacterial communities to reforestation. To achieve this, we conducted a comprehensive field study of soil bacterial community and functions along a vertical profile in a typical artificial forestation with different reforestation ages. We systematically examined the soil bacterial diversity and composition, as well as the dominant functions under artificial forests with different stand ages along a vertical profile (0–10 meters). The outcome of this work is expected to contribute to a more systematic understanding of how reforestation would affect the deep soil bacterial community and functions and provide data support for ecological management practices in the Loess Plateau.

## 2 Materials and methods

### 2.1 Sites and soil sampling

Soil samples were collected in the spring of 2021 from five different forest sites located in the Gutun watershed of the Loess Plateau (Supplementary Figure S1). This area is characterized by a typical continental climate regime and is influenced by East Asian monsoon to different extents. The mean annual temperature is 9.8°C, and the mean annual precipitation is 541 mm (Zhao et al., 2020). The most common vegetation species in the watershed is *Robinia pseudoacacia L.* with a total coverage rate of 60–75%. The field experiment was conducted within the artificial *Robinia pseudoacacia* forests of different stand ages, including R8 (stand age 8 yrs), R22 (stand age 22 yrs), and R32 (stand age 32 yrs). An additional farmland near the forest served as the control F, where sweet potatoes were interplanted with cherry trees. The basic characteristics of the forests and farmland examined are presented in Supplementary Table S1.

Soil samples were collected in each forest and the farmland in April 2021. Samples from 0–200 cm layers were collected at 20 cm intervals, and those from 200–1,000 cm layers were collected at 50 cm intervals using a 5 cm diameter soil. Considering that soils from the upper layers are more susceptible to changes in the environment, while soils in the deeper layers are relatively stable, we categorized them into three levels: 0–200 cm (top layer), 200–500 cm (middle layer), and 500–1,000 cm (deep layer). Soil samples from different layers within each level were considered subsamples for each level. Soil samples were transported to the laboratory in a 4°C esky after sectioning. The soil was sieved through a 2 mm sieve after removing small stones and plant roots. After mixing evenly, soil samples were divided into two parts, with one for the soil physicochemical analysis and the other for soil DNA extraction.

### 2.2 Soil characteristics

Soil water content (SWC) was measured gravimetrically by drying 20 g soil samples overnight in an oven at 105°C. The gravimetric moisture content was then calculated as grams of moisture lost per gram of dry soil. Soil pH was determined using a pH meter at 1:2.5 v:w ratio (Mettler Toledo S210, Mettler-Toledo, Switzerland). Soil organic matter was determined by the potassium dichromate method (H_2_SO_4_-K_2_Cr_2_O_4_) (Walkley and Black, 1934; Alhassan et al., 2018). Soil total nitrogen (TN) was measured by the Kjeldahl digestion method with a Kjeltec analyser (KjeltecTM8400 Analyser, Foss, Sweden) (Bremner, 1960). Soil total phosphorus (TP), available phosphorus (AP), ammonia nitrogen (NH_4_^+^-N), and nitrate-nitrite (NO_3_^−^-N) were measured by a flow injection analyzer (SEAL Analytical AA3, Norderstedt, Germany).

### 2.3 High-throughput sequencing of 16S rRNA genes

Bacterial 16S rRNA genes were amplified using the primer pair 338F (ACTCCTACGGGAGGCAGCAG) and 806R (GGACTACHVGGGTWTCTAAT) (Lee et al., 2012). Sequencing libraries were established using a TruSeqDNA PCR-Free Sample Preparation Kit (Illumina) after purifying PCR products. The quality of the library was initially determined with a Qubit@2.0 Fluorometer (Thermo Scientific) and then Agilent Bioanalyzer 2,100 System (Agilent Technologies). Sequencing analysis was performed on an Illumina MIseq platform (Chen et al., 2019) with a PE250 strategy. Raw reads (2 × 250 bp paired-end) were analyzed mainly through the pipeline of Quantitative Insights into Microbial Ecology (QIIME) (Caporaso et al., 2010). Briefly, chimeras were filtered out using the default program ChimeraSlayer within QIIME (Haas et al., 2011), and reads were clustered into operational taxonomic units (OTUs) using UPARSE at 97% identity (Edgar, 2013). The representative sequences of OTUs were annotated against the latest SILVA database (Quast et al., 2012) using Ribosomal Database Project (RDP) classifier (Wang et al., 2007). Resultant OTU tables were rarefied to a sequencing depth of 20,000 reads per sample before downstream analyses.

Co-occurrence networks of soil bacterial communities in the different levels were constructed using the CoNet app in Cytoscape and visualized by Gephi (Bastian et al., 2009; Faust and Raes, 2016). Briefly, OTUs with relative abundance <0.05% and occurring in less than half of the samples were removed before analysis. Pearson and Spearman correlation, Bray–Curtis dissimilarity, and Kullback–Leibler dissimilarity were used to measure the robustness of the correlation in the network simultaneously (Faust et al., 2015). The *p*-values threshold of the Benjamini-Hochberg’s false discovery rate correlation was 0.001. The topology parameters including average degree, modularity, clustering coefficient, average path length, betweenness centrality, and closeness centrality were calculated to compare the stability and complexity of different networks. PICRUSt2 was used to predict the functions of the soil bacterial communities (Douglas et al., 2020a,b). The nearest sequenced taxon index (NSTI) values were calculated to represent the reliability of the prediction (Douglas et al., 2020b; Canarini et al., 2021).

### 2.4 Statistical analyses

We used the Shannon index to demonstrate the alpha diversity of the bacterial community, as it evaluates both evenness and richness (Gotelli and Colwell, 2001; Wang et al., 2018). Bray−Curtis distance was calculated as a measurement of surface soil layer-to-deep soil layer dissimilarity in bacterial community composition (beta diversity). Principal coordinate analysis (PCoA) was employed on Bray-Curtis distances to visualize the differences in microbial community composition. Moreover, Adonis permutational multivariate analysis of variance (PERMANOVA, Adonis) was conducted to verify the results of PCoA. Distance-based redundancy analysis (db-RDA) was used to identify the effects of soil properties on soil bacterial communities. Variance Inflation Factor (VIF) analysis was performed before RDA to eliminate the effect of multicollinearity; only variables with low VIF (< 10) were kept to perform the RDA. Linear discriminant analysis coupled with effect size (LEfSe) with an LDA score > 3.5 was used to determine taxa with significant differences in abundance among different forests (Segata et al., 2011). Across all abundant taxa in all taxonomic levels of reforestation ages shown in LEfSe analysis, species that showed significant differences in reforestation were known as biomarkers that played important roles in the soil bacterial community of each reforestation age (Segata et al., 2011).

One-way analysis of variance (ANOVA) was used after Levene’s test of homogeneity of variances to detect the differences among soil layers and forests with different stand ages. Spearman correlation was used to determine the relationship between soil properties and bacterial community compositions. Multivariate linear regression was conducted to estimate the driving factors of soil bacterial diversity. Analysis of variance (ANOVA) test of models was conducted. The normality of unstandardized residuals was checked using the Shapiro–Wilk test. The Durbin-Watson test was conducted to examine the autocorrelation of the regression variables. Data points that had a Cook’s distance greater than one indicated that it might be an outlier and should be eliminated (Field, 2009; Neina et al., 2016). All statistical analyses were performed using SPSS 20.0 (IBM Co., Armonk, NY) and R.^1^

## 3 Results

### 3.1 Variations of soil bacterial community diversity

**Figure 1:**
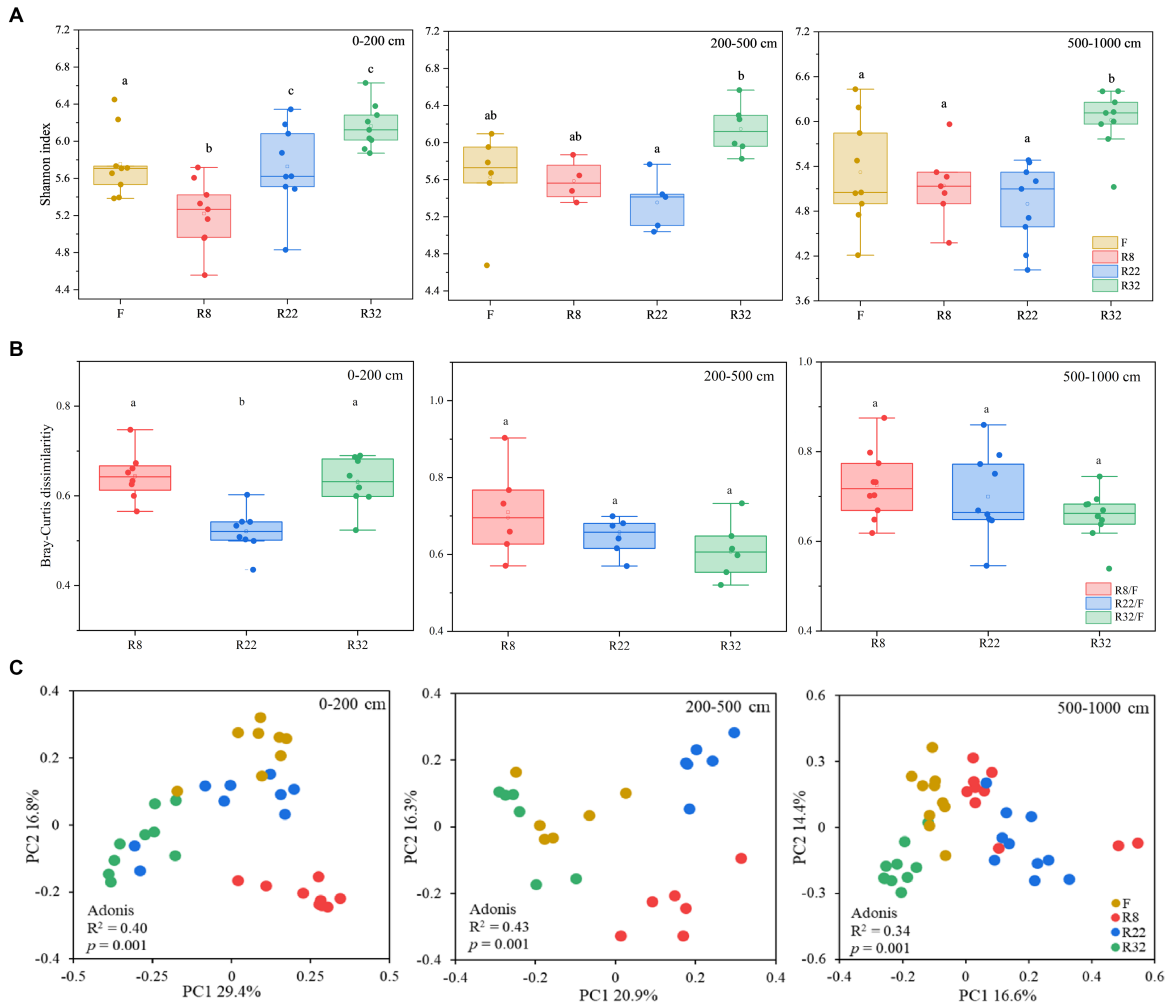
Soil bacterial alpha diversity (Shannon index) of the forests with different reforestation ages as well as the farmland **(A)**. Bray-Curtis distances between forests of different reforestation ages and the control (farmland) at different soil layers **(B)**. Principle coordinates analysis (PCoA) based on the Bray-Curtis matrix of the soil bacterial gene profiles from different forests and farmland **(C)**.

Soil bacterial diversity displayed distinct variations through the reforestation processes across different soil layers. The alpha diversity of soil bacteria, calculated by the Shannon index, showed an upward trend along the reforestation sequences in 0–200 and 200–500 cm soil layers (Figure 1A). No significant difference in alpha diversity could be observed in R8, R22, and F, while alpha diversity in R32 was significantly higher than the others in the 500–1,000 cm soil layer (*p* < 0.05). In all soil layers, R32 consistently exhibited the highest Shannon index values. Soil bacterial alpha diversity in the forest was significantly different than in the farmland at the top layer (0–200 cm) (*p* < 0.05). These results indicate that reforestation led to variations of bacterial alpha diversity in all soil layers, with R32 demonstrating the most substantial diversity across all layers.

To evaluate the changes in soil bacterial community beta diversity after reforestation, the Bary-Curtis distance of soil bacterial communities in different forests relative to the control (farmland) was compared in different soil layers (Figure 1B). In the topsoil layer (0–200 cm), soil bacterial beta diversity in R22 significantly deviated from that of the farmland (*p* < 0.05), while in deep soil layers, there were no significant differences in soil bacterial beta diversity between farmland and forests of different reforestation ages. Variations of soil bacterial community composition were illustrated by PCoA based on the Bray-Curtis distances (Figure 1C). The bacterial community composition in 0–200 cm, 200–500 cm, and 500–1,000 cm layers for R8, R22, R32, and F (the control) appeared as distinct clusters. Adonis analysis further confirmed the distinct bacterial profiles among forests of different reforestation ages in all soil layers (0–200 cm: *R*^2^ = 0.40, *p* = 0.001; 200–500 cm: *R*^2^ = 0.43, *p* = 0.001; 500–1,000 cm: *R*^2^ = 0.34, *p* = 0.001).

### 3.2 Variations of soil bacterial community composition and co-occurrence patterns

**Figure 2:**
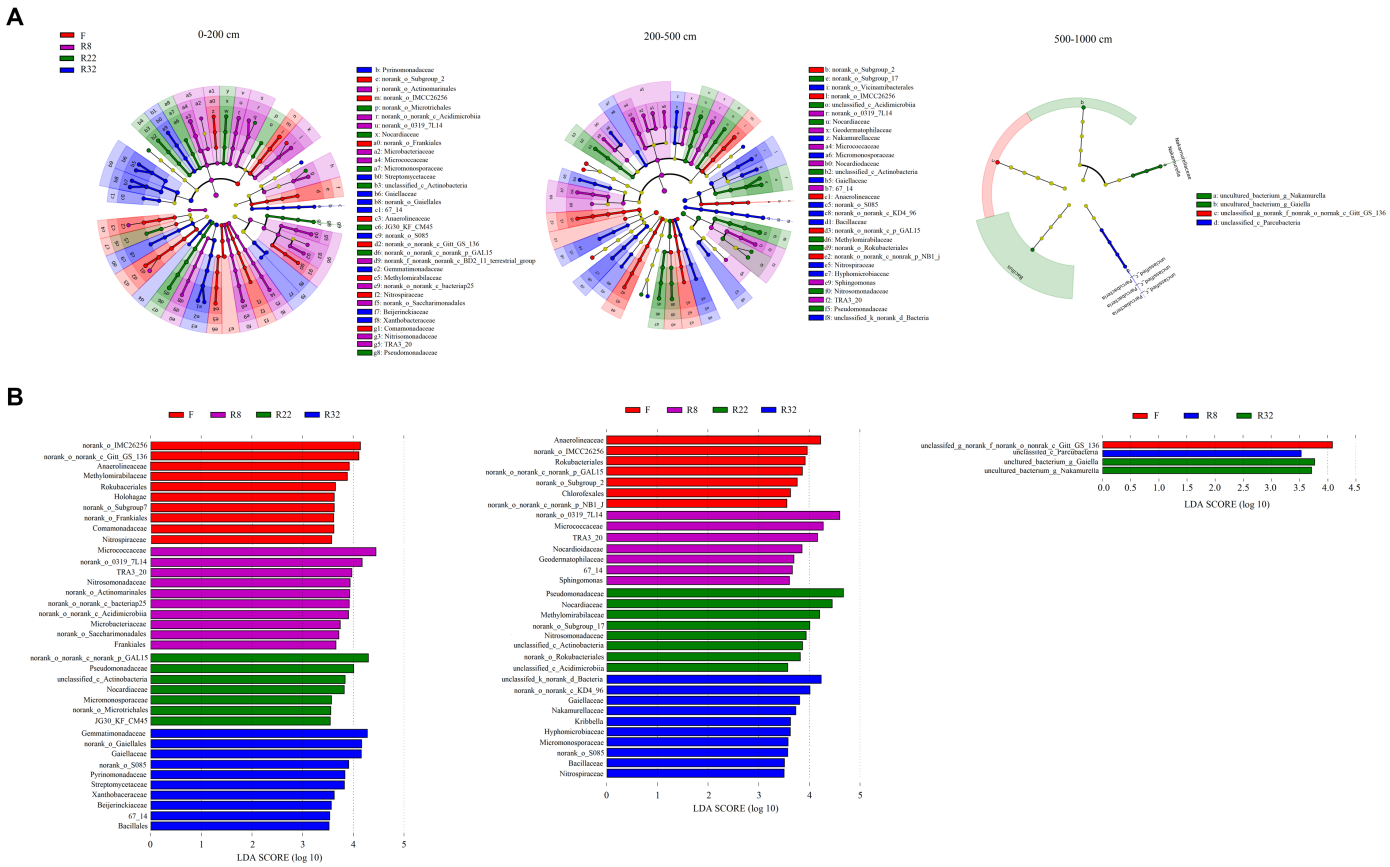
Taxonomic composition of soil bacterial community based on the metagenomes from four soils of different stand ages **(A)**. The non-significantly different taxa are not shown in the cladogram. The dots from the center to the outer sphere represent the phylum, class, order, family, and genus levels. Only taxa at the family level were listed in the legend; the letters were the abbreviation of family-level biomarkers shown in the cladogram correspondingly. Each dot has an effect size LDA score > 3.5. The LDA score identifies the size differentiation among the forests and the farmland at the family level with a threshold score of 3.5 **(B)**.

Results of LEfSe analysis were shown by LDA score and a cladogram visualizing all detected bacterial compositions from phylum to species, respectively (Figure 2). The biomarkers associated with each reforestation age along the soil profiles were dissimilar. A greater number of biomarkers were detected in the topsoil layer (0–200 cm) than in the other layers; at the family level, biomarkers in the largest age of reforestation (R32) were more abundant than the others in each soil layer (Supplementary Table S2). The number of the significant biomarkers at the family level decreased in the deep (500–1,000 cm) soil layer, suggesting that the soil bacterial composition in this soil layer was relatively stable and consistent during the reforestation.

Among all the identified biomarkers, more family-level biomarkers were detected in the topsoil layer, and their abundance declined with increasing soil depth (Supplementary Table S2). Specifically, *Actinobacteriota* were the potential taxonomic indicators that primarily changed under reforestation in the topsoil layer. Families including *Frankiales, Micrococcales*, *Actinomarinals*, and *norank_c_Acidimicrobiia* (LDA 4.66, *p* = 0.003) were significantly abundant in R8. The abundance of *Gammaproteobacteria* (LDA 4.29, *p* = 0.02) including *Burkholderiales* and *TRA3_20* was also significant in R8. Fewer biomarkers can be detected in the middle soil layer (200–500 cm) than the topsoil layer (0–200 cm). *Actinobacteriota* and *Proteobacteria* were the main phyla that contained potential biomarkers in the middle soil layer. *Actinobacteriota* including *Geodermatophilaceae*, *Micrococales*, *Nocardioidaceae*, and *TRA3_20* enriched in R8 (LDA 4.85, *p* = 0.006), while *Gammaproteobacteria* was more abundant in R22 (LDA 4.76, *p* = 0.001). In the deep soil layer (500–1,000 cm), the order *norank_c_Gitt_GS_136* (phylum *Chloroflexi*) was abundant in F (LDA 4.08, *p* = 0.000), and *Unclassified_c_Parcubacteria* (phylum *Patescibacteria*) was more abundant in R8 (LDA 3.53, *p* = 0.000). The families *Nakamurellaceae* (phylum *Actinobacteriota*) and *Bacillaceae* (phylum *Firmicutes*) were significantly enriched in R32 with an LDA score of 3.725 and 3.619, respectively (*p* = 0.000).

**Figure 3:**
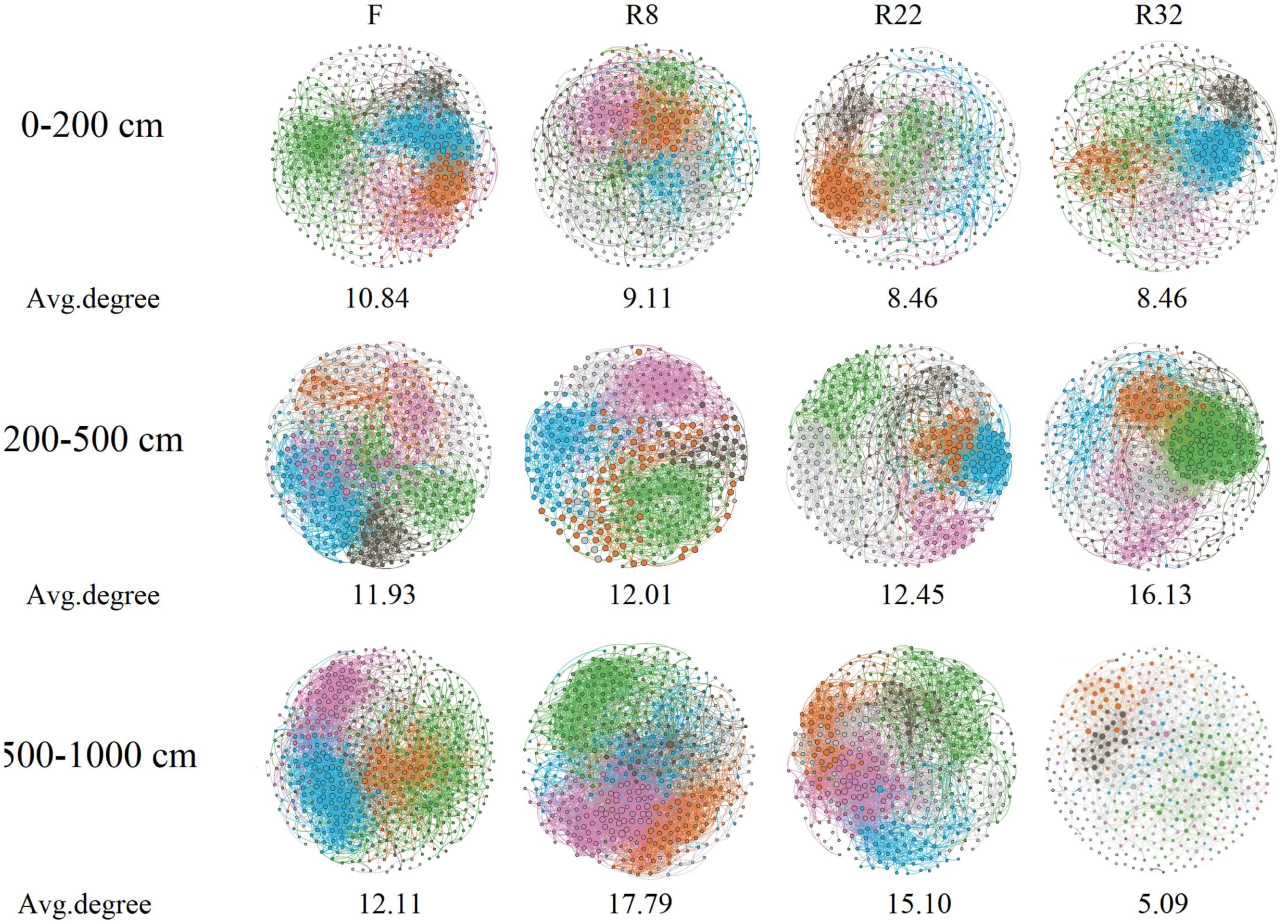
Bacterial co-occurrence networks in forests of different reforestation ages and farmland at different soil layers. The average degree of each network is shown in the figure.

Correlation networks among bacterial OTUs were constructed to access the soil bacterial co-occurrence patterns in the forest with different ages of reforestation along the vertical profile. Results demonstrated that the complexity and the connection of soil bacterial communities were dissimilar (Figure 3). Specifically, the average degree of the network of F was higher than that in the forest with different reforestation ages at the 0–200 cm soil layer. In the 200–500 cm layer, the average degree of R32 was the highest, but the modularity was the lowest (0.49) (Supplementary Table S3). Interestingly, the edge and the average degree of each network increased as the soil depth increased to 500–1,000 cm except R32, while the average path length decreased by increasing the soil depth except R32.

### 3.3 Variations of soil bacterial functions

**Figure 4:**
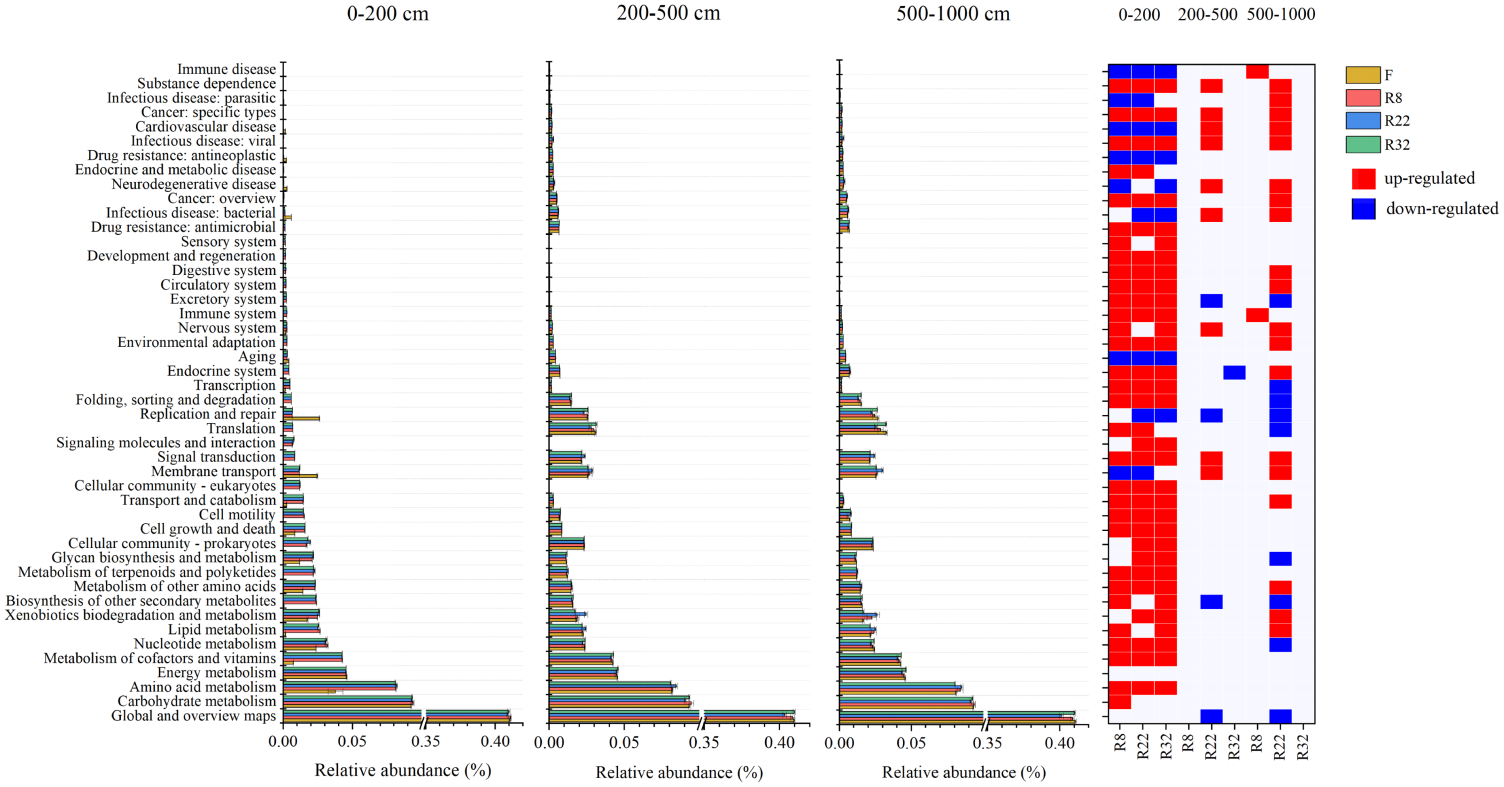
KEGG annotations of predicted functions of all samples. Relative abundance of 46 functions in KEGG pathway level 2 in different soil layers. Significant changes in the relative abundance of predicted functions induced by reforestation in different soil layers. Red represents functions that are enriched by reforestation; blue represents functions that are depleted by reforestation.

The relative abundance of functional groups in KEGG pathway level 2 predicted by PICRUSt2 differed between forests and farmland (Figure 4). The average weighted nearest sequenced taxon index (NSTI) for all samples was 0.12, falling within the conventional soil threshold range, indicating a moderate accuracy of the functional prediction (Langille et al., 2013; Douglas et al., 2020b). Across all treatments, the top three function traits in bacterial communities were global and overview maps, carbohydrate metabolism, and amino acid metabolism. Reforestation induced enrichment in most functional pathways, particularly in the 0–200 cm soil layer (Figure 4). In this layer, 31, 30, and 32 functional pathways increased in R8, R22, and R32, respectively, compared to the farmland, while seven, eight, and seven functional pathways decreased in the respective forest. In the 200–500 cm soil layer, nine functional pathways increased and three functional pathways decreased in R22, and only one functional pathway decreased in R32, while in the 500–1,000 cm soil layer, two functional pathways increased in R8 and nineteen increased and eight functional pathways decreased in R32. Specifically, amino acid metabolism increased in the 0–200 cm soil layer due to reforestation, while pathways involved in secondary metabolites biosynthesis decreased in R22 in the 200–1,000 cm soil layers.

### 3.4 Factors driving soil bacterial taxonomic and functional composition

**Figure 5:**
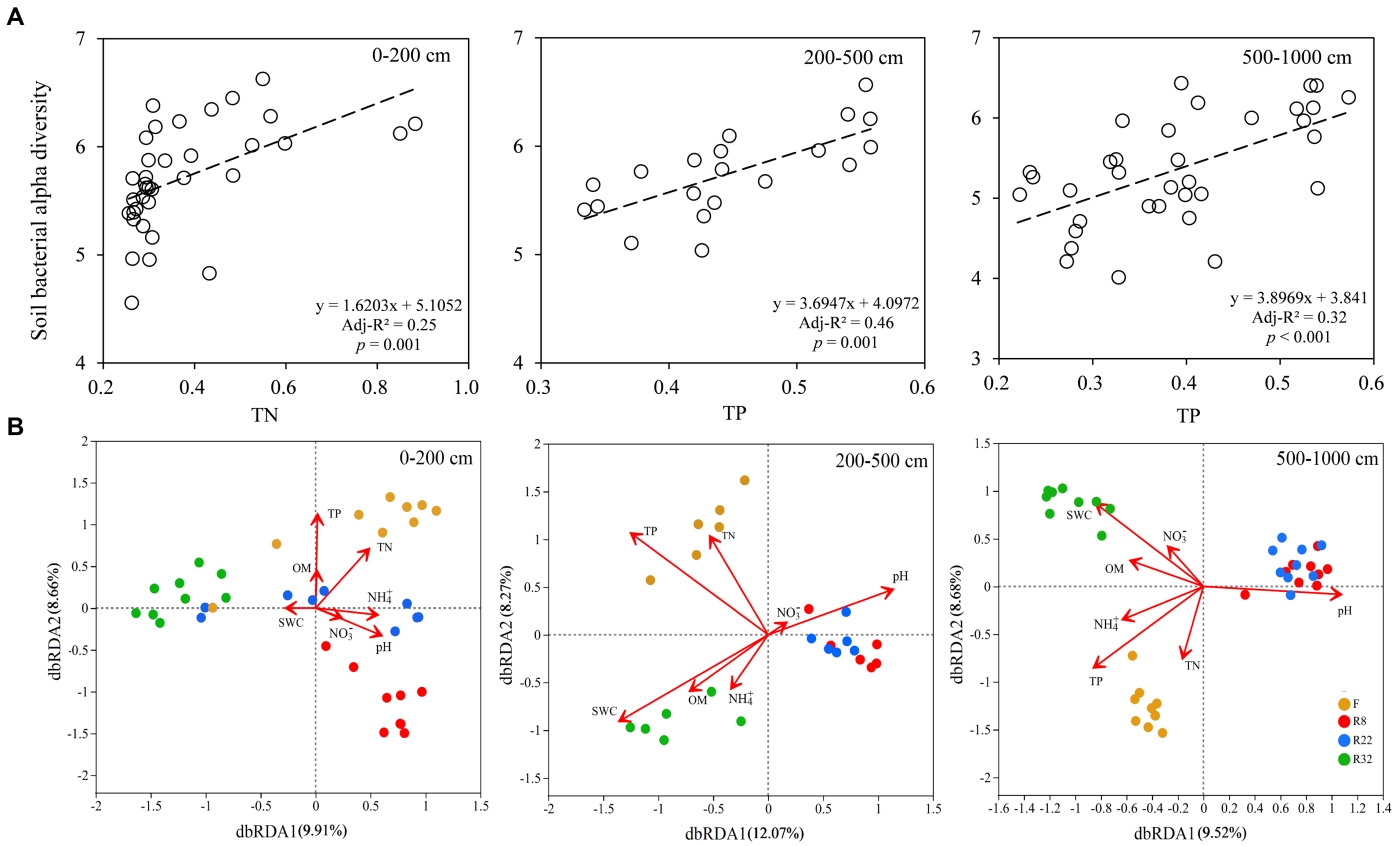
Linear regressions of soil bacterial alpha diversity (Shannon index) and soil total phosphorus (TP) along the reforestation in the top (0–200 cm), middle (200–500 cm), and deep (500–1,000 cm) soil layer **(A)**. Distance-based redundancy analysis (db-RDA) of soil bacterial communities during reforestation in the top (0–200 cm), middle (200–500 cm), and deep (500–1,000 cm) soil layer **(B)**. The ordination is based on the Bray-Curtis distance. Red arrows indicate the environmental variables and dots of different colors represent soil bacterial communities (OTU-level) in different successional stages of reforestation. TP: soil total phosphorus; TN: soil total nitrogen; OM: soil organic matter; NH_4_^+^: soil ammonium nitrogen content; NO_3_^−^: soil nitrate nitrogen content; SWC: soil water content.

In the topsoil layer, the linear regression model showed that soil total nitrogen (TN) was the most important factor regulating bacterial alpha diversity along the reforestation sequence, while soil total phosphorus (TP) was the main factor driving alpha diversity along the reforestation sequence in the deep soil layer (Figure 5A). Distance-based redundancy analysis (db-RDA) on the OTU level showed that soil properties explained 18.57% of the variability in the community in the topsoil layer, where TP, TN, and soil pH were the most important factors. In the middle soil layer, the soil main properties explained 20.34 and 18.2% in the middle and deep soil layer, respectively (Figure 5B); TP, SWC, and soil pH were the most influential factors driving soil bacterial community along reforestation in the two layers.

**Figure 6:**
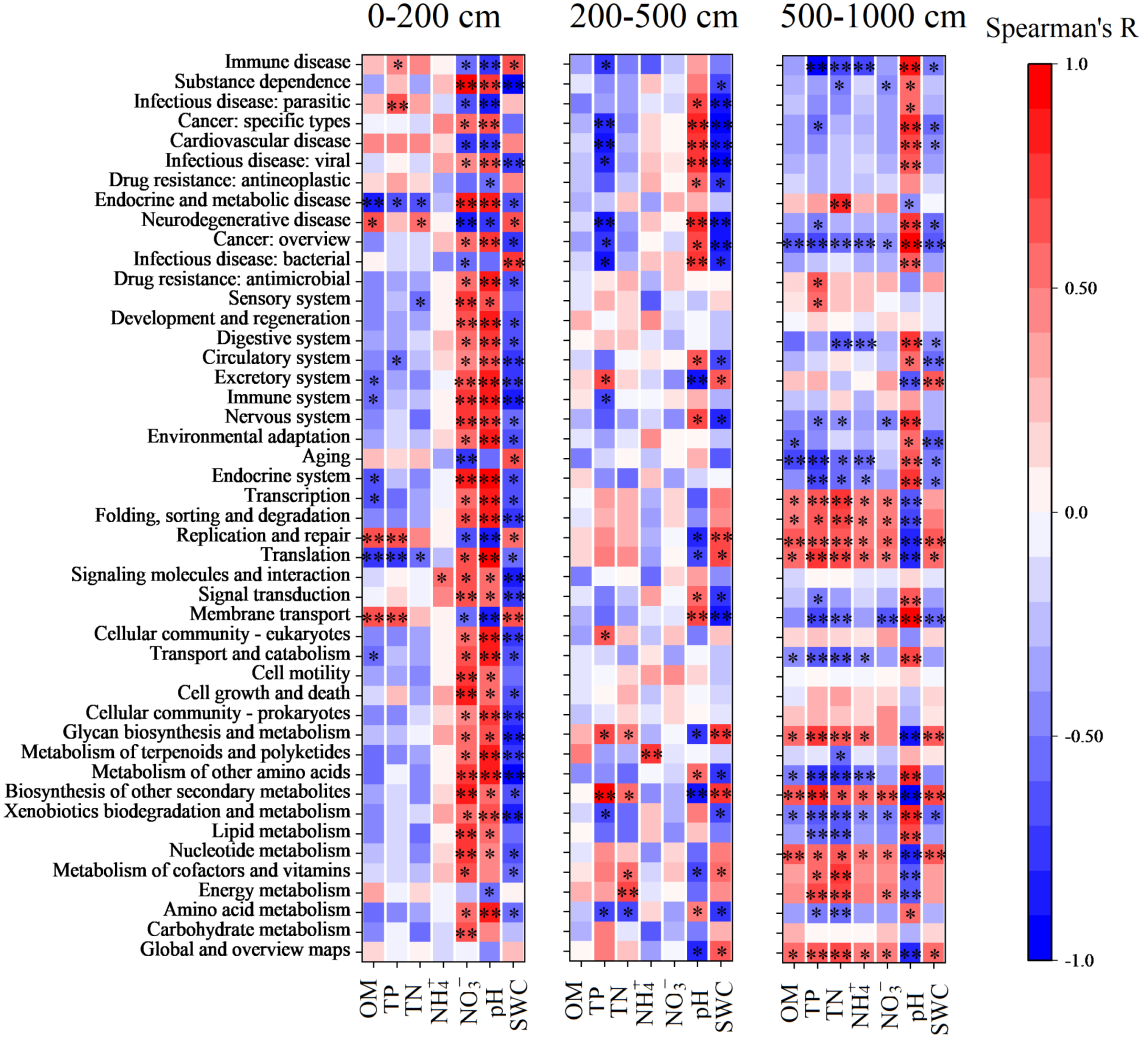
Spearman’s correlation between predicted functions and soil physicochemical properties in the top (0–200 cm), middle (200–500 cm), and deep (500–1,000 cm) soil layers. Asterisks represent significant levels of correlation. *p < 0.05, **p < 0.01.

In addition, the major functions of soil bacteria were significantly correlated with SWC, soil pH, and soil NO_3_^−^ content in the topsoil layer; the major functions of soil bacteria were significantly affected by soil pH, SWC, and soil TP in the middle and deep soil layers, while in the deep soil layer, soil bacteria functions were mainly affected by nutrients content and SWC and soil pH (Figure 6).

## 4 Discussion

### 4.1 Effects of reforestation on soil bacterial communities along the vertical profile

Soil bacterial diversity was largely related to soil nutrients (Meisser et al., 2019). In the topsoil layer, bacterial alpha diversity was promoted by the increased soil TN content. Plant litter provides sufficient nutrients for soil bacteria in the topsoil layer (Li H. et al., 2019; Li Y. B. et al., 2019). Nitrogen content was an important control factor of litter decomposition and utilization by bacteria (Zhang et al., 2008). Sufficient water content could promote nitrogen mineralization processes, thus nutrient limitation may be raised in the dry condition. In the deep soil of the farmland, soil TP significantly negatively altered the soil bacterial alpha diversity since the abundant usage of phosphate fertilizer and high degree of water saturation (average SWC of 14.38% in 1000–1,500 cm) led in turn to greater P leaching (Keshavarzi et al., 2018; Yan et al., 2018; Khan et al., 2019).

According to the distance-based redundancy analysis (db-RDA) results, nutrient elements were dominant driving factors of soil bacterial community compositions in reforestation as the soil bacteria relied on the content and availability of nutrients in the soil (Shen et al., 2019). Studies have proved that reforestation altered the distribution and stocks of soil TN, TP, and SOC (Wang et al., 2014; Veldkamp et al., 2020). In addition, soil bacterial communities participated in the processes of soil organic matter decomposition and altered the availability of nutrients (Cui et al., 2018, 2020). Meanwhile, nutrient content and availability could lead to variations in soil bacterial interactions and community responses, as most of the soil bacteria groups are heterotrophic (Zechmeister-Boltenstern et al., 2015; Tian et al., 2018; Soong et al., 2020).

Previous studies have reported that forest stand age can lead to significant changes in soil hydrological properties (Zema et al., 2021). As an indicator of reforestation status, forest stand age would contribute to soil water fluctuations, which can influence soil bacterial activities along the vegetation succession (Cui et al., 2020). In this study, the water content of F was significantly higher than that of the forest of reforestation in the deep soil layer (Supplementary Figure S2). Plant roots mediated soil water content and distribution by root water uptake (Rummel et al., 2021). Studies have shown that reforestation would deplete deep soil water since plant roots can uptake deep soil water to support their growth, leading to deep soil water deficits (Kong et al., 2022). With the increase in forest stand age, plant root depth increases, and the process of root uptake of deep soil water is more likely to occur (Tao et al., 2021). However, this process occurs only when the water content of deep soil is sufficient, which in turn affects the growth and activity of bacteria in the soil (Li H. et al., 2019; Li Y. B. et al., 2019). Resource competition, such as nutrients and water, between plants and soil bacteria may lead to changes in soil bacterial community composition. The changes were related to the condition of vegetation growth and root absorption processes of water and nutrients (Guyonnet et al., 2018b). The nutrient uptake strategies of plants could alter the carbon output by releasing carbon into the soil via root exudation, which could therefore influence the soil bacterial community composition and functions (Guyonnet et al., 2018a).

In addition, the connections among soil bacterial species varied among different forest and soil layers. The node and edge of the co-occurrence networks in the topsoil layer decreased along the reforestation (Supplementary Table S3), indicating reforestation resulted in less association in bacteria in the topsoil layer. In addition, reforestation decreased the average connectivity (average degree) of the network by 21.99% in the topsoil layer. However, reforestation increased the average connectivity of the network by 35.16% in the middle soil layer, while the average connectivity increased at first but decreased sharply (57.92%) by the end of the reforestation in the deep soil layer. Reforestation largely reduced the deep soil layer network node by 14.81–21.94%. The network modularity of the top and deep layers was enhanced by 2.73 and 17.98%, respectively, while the reforestation decreased the network modularity by 19.94% in the middle soil layer. The average clustering coefficient of the network in the top and deep layers was decreased by 1.14 and 21.96%, respectively, while the reforestation increased the average clustering coefficient of the network by 2.64% in the middle soil layer. Reforestation increased the average path length of networks in all soil layers by 9.38, 22.87, and 37.26%, respectively. An increase in average path length by reforestation could reduce the response of the network to environmental perturbations and induce a more loosely connected bacterial community (Faust and Raes, 2012). In general, reforestation resulted in a less complex and less hierarchical network with higher modularity and looser structure in the top and deep soil layers, while in the middle soil layer, reforestation led to a less complex and more hierarchical network with higher modularity and looser structure (Deng et al., 2012; Prescott et al., 2022).

Reforestation increased the relative abundance of the great majority of soil bacterial functional pathways. Metabolism was the most abundant function of all. The variation of metabolism pathways in forests with different reforestation ages indicates that reforestation can lead to functional trait fluctuation. In the topsoil layer, reforestation resulted in enrichment in relative abundance of most functional pathways. Specifically, an abundance of the carbohydrate metabolism pathway was up-regulated in forests of all reforestation ages. Higher carbohydrate metabolism could provide more substrates by degrading cellulose and hemicellulose (López-Mondéjar et al., 2016). The energy released during carbohydrate metabolism in microorganisms sustains bacterial growth while maintaining functional cellular homeostasis (Cao et al., 2021). In the deep soil layer, the carbohydrate metabolism pathway was insensitive to reforestation suggesting that the regulatory effects of reforestation on soil bacterial functions, especially metabolism pathways, were non-significant.

### 4.2 Effects of soil depth on soil bacterial in forests of different reforestation age

Soil bacterial beta diversity was mainly driven by soil TP and TN in the topsoil layer (Figure 5). This may have resulted from the variation of soil elemental factors along the reforestation (Supplementary Figure S3) since the majority of soil bacteria obtained energy (essential nutrients) from the decomposition and mineralization of soil organic matter (Trivedi et al., 2013). The surface soil possessed high soil nutrient contents (e.g., TN) derived from the accumulation of organic residues of surface litter and root exudates; the abundant nutrients were tightly correlated to soil bacterial diversity by affecting the bacterial growth and metabolism (Fierer et al., 2003; Trivedi et al., 2013; Delgado-Baquerizo et al., 2017; Prescott and Vesterdal, 2021). However, as these properties decreased with soil depth, their effects on soil bacterial diversity also decreased gradually. Deeper layers showed less water and nutrients as well as less available O_2_ for soil bacteria to grow (Li et al., 2016; Cai et al., 2020); thus, the soil bacterial communities in deeper soil layers were less abundant and diverse. Lower contents of nutrients in deeper soil layers resulted in the limitation for soil bacteria to grow, thus leading to smaller microbial densities and diversity (Delgado-Baquerizo et al., 2016). Soil TP was significantly positively correlated with soil alpha diversity and it was the most significant driving factor of bacterial beta diversity. The availability of phosphorus was largely related to the bedrock; thus soil TP was the main driving factor of soil bacterial abundance and richness in the deep soil layer (500–1,000 cm). Furthermore, soil pH played an important role in driving soil bacterial beta diversity in all soil layers which was consistent with previous studies that confirmed that pH was a universal driving factor of soil bacterial diversity and community composition (Fierer and Jackson, 2006; Singh et al., 2012; Delgado-Baquerizo and Eldridge, 2019; Li et al., 2021). The soil pH in deeper layers was higher than that in the top layers, but as the soil of the Loess Plateau is alkaline, the increase of soil pH in the vertical direction may not lead to the fluctuation of the soil bacterial community (Chu et al., 2010; Liu et al., 2013; Jin et al., 2019).

With the increase in soil depth, the significantly different taxa of soil bacterial community composition in the soil of the forest of different reforestation ages gradually decreased (Figure 2; Supplementary Table S2). The biomarkers of each forest were changed simultaneously, which could be used to distinguish soil bacterial communities of different treatments (Segata et al., 2011). The soil bacterial communities in deeper soil layers contained fewer biomarkers that were selected by the deep soil environment (Fierer et al., 2003). It was the environmental variations within the soil profile that caused soil bacterial community composition changes along the soil depth.

Among all the family-level biomarkers, the order *Saccharimonadales* belonged to the candidate phylum *Saccharibacteria* which was formerly known as *TM7*. *Saccharibacteria* have been proven to survive by co-metabolizing with other bacteria as they have small cell sizes and genomes (Lemos et al., 2019). As a specific biomarker in the 0–500 cm soil layers of R8, *Saccharimonadales* was significantly altered by SWC in the 200–500 cm soil layer. Their abundance was enriched in R8 since the SWC was relatively high in the 0–500 cm soil layer. Many groups of *Saccharibacteria* could utilize complex carbon sources and degrade into carbon sources through a co-metabolism process in order to promote their growth and those of other bacteria in soil (Rüthi et al., 2020), thus stimulating the soil bacterial community in the young forest. The family *Moraxellaceae* is the biomarker in 1000–1,500 cm of R22, which belongs to *Gammaproteobacteria*; certain species are mesophilic or psychrotrophic. The order-level biomaker *Rokubacteriales* in the 40–500 cm soil layers of R32 were affected by SWC and soil nutrients (OM, TN, and TP). *Rokubacteriales* is an order of *Rokubacteria*, that has the potential for nitrogen respiration, sulfur oxidation, and sulfate/sulfite reduction (Becraft et al., 2017; Anantharaman et al., 2018). In the over-mature forest, the variation of soil properties may influence the nitrogen- and sulfur-related processes by altering the abundance of *Rokubacteriales*.

## 5 Conclusion

Overall, this study highlights the profound regulator effect of reforestation on soil bacterial community structure, especially in the deep soil layer. Reforestation increased bacterial alpha diversity and the number of biomarkers and resulted in a less complex, more distant, and less hierarchical network with higher modularity in both top and deep layers. Deep soil bacterial functions were enriched by reforestation with an age of 22, highlighting the significance of reforestation age as a key factor influencing soil bacterial community responses. The responses of soil bacterial communities to reforestation vary with soil depth, with soil bacterial community structure being notably influenced by increasing soil depth. The findings of this work provide insight into the responses of deep soil bacterial community structure to ecological reforestation which is crucial for studying the deep layer of the terrestrial ecosystem response to human-induced environmental changes.

## Data availability statement

The data presented in the study are deposited in the NCBI repository, accession number PRJNA1033595.

## Author contributions

FW: Conceptualization, Data curation, Investigation, Methodology, Software, Writing – original draft, Formal analysis, Funding acquisition, Validation, Writing – review & editing. YW: Conceptualization, Supervision, Writing – review & editing, Validation. HS: Software, Visualization, Writing – review & editing, Data curation. JZ: Data curation, Investigation, Writing – review & editing, Formal analysis. RL: Formal analysis, Investigation, Writing – review & editing.

## Footnotes

^1^ www.r-project.org
