# Supplementary material for: Reforestation regulated soil bacterial community structure along vertical profiles in the Loess Plateau
Source: Front Microbiol. 2023 Nov 28;14:1324052. doi: 10.3389/fmicb.2023.1324052 (PMC10713748; doi:10.3389/fmicb.2023.1324052)
Supplement: Supplementary file 1 [file Data_Sheet_1.docx]

***Supplementary Material***

# Reforestation regulated soil bacterial community structure along vertical profiles in Loess Plateau

**Fan Wu^1,2*^, Yunqiang Wang^2,3*^, Hui Sun^3,4^, Jingxiong Zhou^2,3^, Ruijie Li^3,4^**

^1^Key Laboratory of Ecosystem Network Observation and Modeling, Institute of Geographic Sciences and Natural Resources Research, Chinese Academy of Sciences, Beijing 100101, People’s Republic of China

^2^University of Chinese Academy of Sciences, Beijing 100049, People’s Republic of China

^3^State Key Laboratory of Loess and Quaternary Geology, Institute of Earth Environment, Chinese Academy of Sciences, Xi'an, Shaanxi 710061, China

^4^Xi’an Institute for Innovative Earth Environment Research, Xi’an 710061, China

**Supplementary Figures and Tables**

**Supplementary Tables**

**Table S1**. Basic characteristic of the forests and farmland examined.

| NO. | Landuse type | Stand Age (year) | Coordinates | Altitude (mm) | Tree type |
| --- | --- | --- | --- | --- | --- |
| R8 | Artificial forest | 8 | 109°49′14″;36°47′28″ | 1171 | *Robinia pseudoacacia L.* |
| R22 | Artificial forest | 22 | 109°47′18″;36°48′56″ | 1108 | *Robinia pseudoacacia L.* |
| R32 | Artificial forest | 32 | 109°48′15″;36°46′32″ | 1092 | *Robinia pseudoacacia L.* |
| F | Farmland | 20 | 109°48′22″;36°46′58″ | 1039 | *Sweet potatoes interplanted with cherry trees* |

R8, R22, and R32 represents the artificial *Robinia pseudoacacia* forests of different stand age, R8 (stand age 8 yrs), R22 (stand age 22 yrs), R32 (stand age 32 yrs); F represents farmland of sweet potatoes interplanted with cherry trees.

**Table S2.** Biomarkers at family level of each treatment.

|  | F | R8 | R22 | R32 |
| --- | --- | --- | --- | --- |
| 0-200 cm | *Anaerolineaceae* | *Frankiales* | *Nocardiaceae* | *Pyrinomonadaceae* |
|  | *Methylomirabilaceae* | *Microbacteriaceae* | *Micromonosporaceae* | *Streptomycetaceae* |
|  | *Rokubacteriales* | *Micrococcaceae* | *JG30_KF_CM45* | *Gaiellaceae* |
|  | *Nitrospiraceae* | *Nitrisomonadaceae* | *Pseudomonadaceae* | *67_14* |
|  | *Comamonadaceae* | *TRA3_20* | *norank_o_Microtrichales* | *Bacillales* |
|  | *norank_o_Subgroup 7* | *norank_o_Actinomarinales* | *unclassified_c_Actinobacteria* | *Gemmatimonadaceae* |
|  | *norank_o_IMCC26256* | *norank_o_norank_c_Acidimicrobiia* | *norank_o_norank_c_norank_p_GAL15* | *Beijerinckiaceae* |
|  | *norank_o_Frankiales* | *norank_o_0319_7l14* |  | *Xanthobacteraceae* |
|  | *norank_o_norank_c_Gitt_GS_136* | *norank_f_norank_o_norank_c_BD2_11_terrestrial_group* |  | *norank_o_Gaiellales* |
|  |  | *norank_o_norank_c_bacteriap25* |  | *norank_o_S085* |
|  |  | *norank_o_Saccharimonadales* |  |  |
| 200-500 cm | *Anaerolineaceae* | *Geodermatophilaceae* | *Nocardiaceae* | *Nakamurellaceae* |
|  | *Chloroflexales* | *Micrococcaceae* | *Methylomicabilaceae* | *Micromonosporaceae* |
|  | *Rokubacteriales* | *Nocardioidaceae* | *Nitrosomonadaceae* | *Kribbella* |
|  |  | *67_14* | *Pseudomonadaceae* | *Gaiellaceae* |
|  |  | *Sphingominas* |  | *Bacillaceae* |
|  |  | *TRA3_20* |  | *Nitrospirales* |
|  |  |  |  | *Hyphomicrobiaceae* |
| 500-1000 cm | *unclassified_g_norank_norank_f_norank_o_norank_c_Gitt_GS_136* | *unclassifed_c_Parcubacteria* |  | *uncultured_bacteriun_g_Nakamurella* |
|  |  |  |  | *uncultured_bacterium_g_Gaiella* |

**Table S3.** Topological properties of soil bacterial co-occurrence networks

| Network properties | Soil levels | F | R8 | R22 | R32 |
| --- | --- | --- | --- | --- | --- |
| Node | 0-200 | 457 | 464 | 417 | 416 |
|  | 200-500 | 443 | 396 | 414 | 458 |
|  | 500-1000 | 547 | 466 | 427 | 468 |
| Edge | 0-200 | 2477 | 2113 | 1763 | 1763 |
|  | 200-500 | 2643 | 2377 | 2578 | 3693 |
|  | 500-1000 | 3311 | 4144 | 3223 | 1192 |
| Average degree | 0-200 | 10.84 | 9.11 | 8.46 | 8.46 |
|  | 200-500 | 11.93 | 12.01 | 12.45 | 16.13 |
|  | 500-1000 | 12.11 | 17.79 | 15.10 | 5.09 |
| Modularity | 0-200 | 0.55 | 0.564 | 0.571 | 0.565 |
|  | 200-500 | 0.617 | 0.612 | 0.643 | 0.494 |
|  | 500-1000 | 0.545 | 0.48 | 0.512 | 0.643 |
| Density | 0-200 | 0.024 | 0.02 | 0.02 | 0.02 |
|  | 200-500 | 0.027 | 0.03 | 0.03 | 0.03 |
|  | 500-1000 | 0.022 | 0.038 | 0.035 | 0.011 |
| Average Clustering Coefficient | 0-200 | 0.351 | 0.315 | 0.347 | 0.347 |
|  | 200-500 | 0.454 | 0.436 | 0.471 | 0.466 |
|  | 500-1000 | 0.337 | 0.382 | 0.436 | 0.263 |
| Average path length | 0-200 | 4.072 | 4.064 | 4.454 | 4.454 |
|  | 200-500 | 3.87 | 3.761 | 4.038 | 4.755 |
|  | 500-1000 | 3.645 | 3.167 | 3.326 | 5.003 |

**Supplementary Figures**

**Figure S1.** The studying area and sampling site. R8, R22, and R32 represents the artificial *Robinia pseudoacacia* forests of different stand age, R8 (stand age 8 yrs), R22 (stand age 22 yrs), R32 (stand age 32 yrs); F represents farmland of sweet potatoes interplanted with cherry trees as the control in the Gutun watershed.


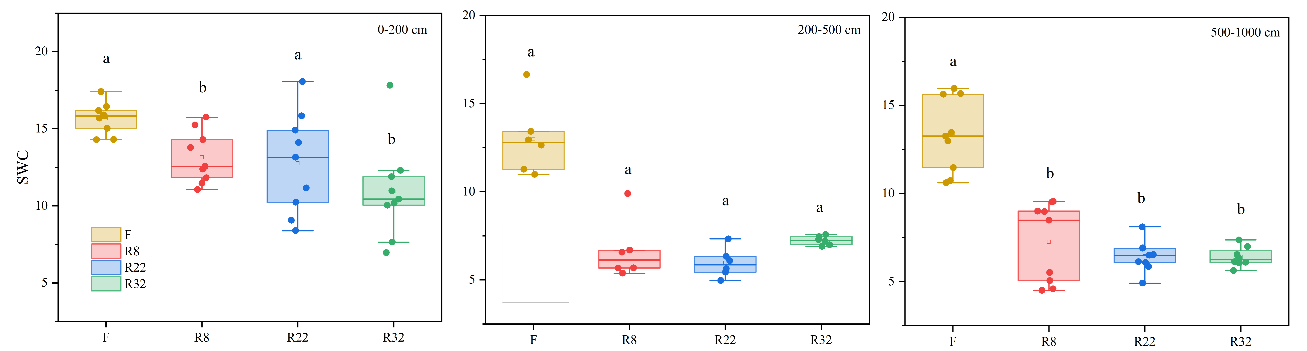


**Figure. S2.** Soil water content (SWC) of forest with different reforestation age and the farmland in different soil layers.


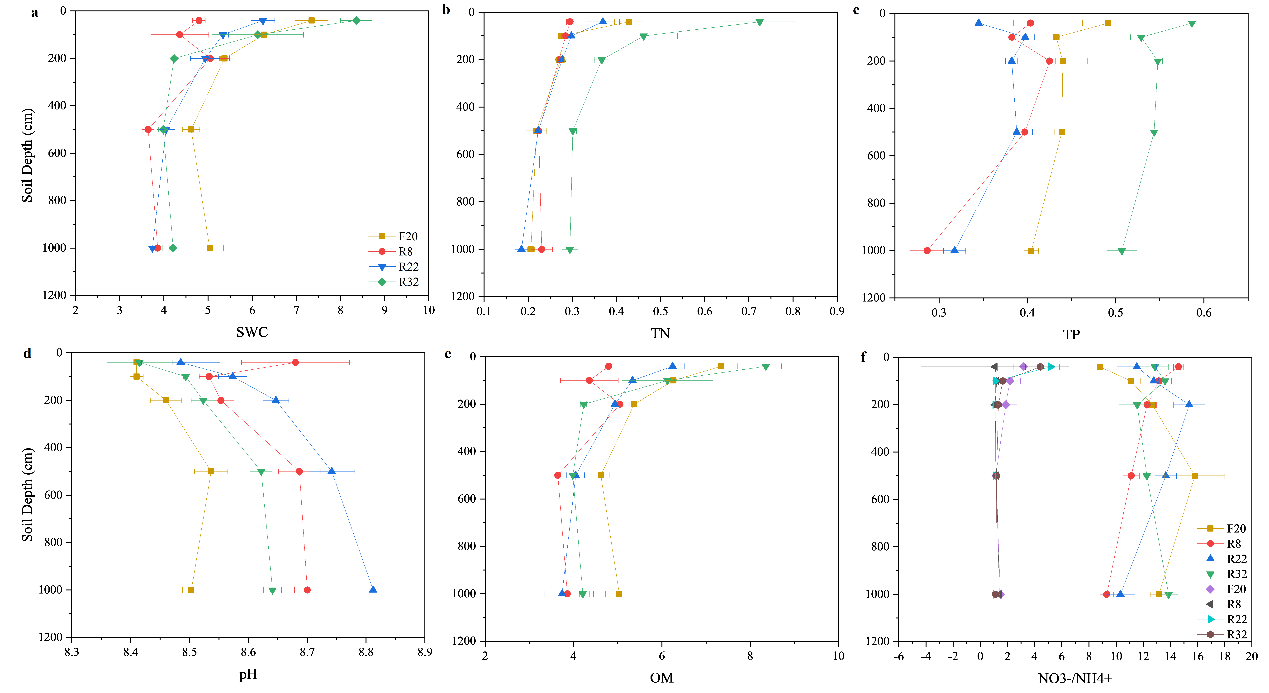


**Figure. S3.** Vertical distribution of soil main properties of forests with different reforestation age (R8, R22, R32) and the farmland (F). a, SWC: soil water content, b, TN: total nitrogen; c, TP: total phosphorus, d, pH; e, OM: organic matter; f, NH_4_^+^: ammonium nitrogen; NO_3_^-^: nitrate nitrogen.
